# Gendered Pathways to Missed and Zero-Dose Polio Vaccination in Children: A Narrative Review of Women’s Autonomy, Household Decision-Making, Structural Barriers, and Health System Responsiveness in Afghanistan, Nigeria, Pakistan, and Sudan

**DOI:** 10.3390/vaccines14080652

**Published:** 2026-07-24

**Authors:** Godfrey Musuka, Patrick Gad Iradukunda, Malizgani Mhango, Oscar Mano, Roda Madziva, Helena Herrera, Noah Mataruse, Tafadzwa Dzinamarira

**Affiliations:** 1 International Initiative for Impact Evaluation, Harare P.O. Box 0002, Zimbabwe; 2 Rwanda Food and Drug Authority, Kigali P.O. Box 1948, Rwanda; 3 School of Public Health, University of Western Cape, Bellville 7535, South Africa; 4 Africa Centre for Inclusive Health Management, Economic and Management Sciences (EMS) Faculty, University of Stellenbosch, Stellenbosch 7602, South Africa; 5 School of Sociology and Social Policy, University of Nottingham, Nottingham NG7 2RD, UK; 6 School of Medicine, Pharmacy and Biomedical Sciences, University of Portsmouth, Portsmouth PO1 2UP, UK; 7 United Nations Children’s Fund, 2150 Copenhagen, Denmark; 8 School of Health Systems and Public Health, University of Pretoria, Pretoria 0002, South Africa

**Keywords:** polio, zero dose, missed children, Nigeria, Sudan, Afghanistan, Pakistan

## Abstract

**Background:** Missed and zero-dose children remain major barriers to polio eradication in settings affected by insecurity, poverty, weak health systems, and social exclusion. This narrative review synthesized evidence on how gender norms and related social determinants influence childhood polio vaccination in Afghanistan, Nigeria, Pakistan, and Sudan. Afghanistan and Pakistan remain the only countries with endemic wild poliovirus transmission, while northern Nigeria, especially areas affected by insurgency, and war-affected Sudan continue to report substantial numbers of polio zero-dose and under-immunized children, together with occasional reports of circulating vaccine-derived poliovirus. **Methods:** A comprehensive search of peer-reviewed and institutional literature was conducted in major databases and relevant grey literature sources. Eligible studies examined childhood immunization or polio vaccination among children under five and reported gender-related determinants of vaccination uptake. Findings were synthesized thematically. **Results:** Forty-one studies were included. Five interconnected domains emerged: women’s empowerment, autonomy, and household decision-making; male involvement, household gender norms, and family power relations; socioeconomic and structural gender-related barriers; education, information access, community perceptions, and health system responsiveness; and war, insurgency and their gendered impact on childhood polio immunization. Across countries, limited maternal autonomy, restricted mobility, dependence on male decision-makers, poverty, conflict, displacement, misinformation, and weak health services reduced access to vaccination. Supportive male engagement, female health workers, trusted community leadership, and culturally responsive outreach facilitated vaccine acceptance. **Conclusions:** Gender influences childhood polio vaccination through intersecting household, community, structural, and health-system pathways. Family power extends beyond male decision-making, with women’s education and influence within households shaping vaccination decisions. Strengthening women’s agency, constructively engaging men (e.g., through husband schools), and delivering culturally responsive services are essential for reaching missed and zero-dose children and accelerating polio eradication.

## 1. Introduction

Poliomyelitis remains one of the most significant vaccine-preventable diseases targeted for global eradication. Although substantial progress has been achieved over the past three decades, transmission continues to occur in a limited number of settings characterized by persistent inequities in vaccination coverage, fragile health systems, insecurity, and social barriers to healthcare access [1]. The emergence and persistence of missed and zero-dose children represent major obstacles to achieving and sustaining polio eradication [2]. Missed children are those who fail to receive vaccination opportunities despite the availability of immunization services, while zero-dose children are those who have not received any routine childhood vaccinations [3]. These populations remain critical reservoirs of vulnerability that threaten both national immunization goals and global eradication efforts.

Afghanistan, Nigeria, Pakistan, and Sudan continue to face substantial challenges in reaching all eligible children with lifesaving vaccines [4,5]. Afghanistan and Pakistan remain the only countries with endemic wild poliovirus transmission, while northern Nigeria, especially areas affected by insurgency, and war-affected Sudan continue to report substantial numbers of polio zero-dose and under-immunized children, together with occasional reports of circulating vaccine-derived poliovirus. Across these countries, armed conflict, population displacement, geographic isolation, socioeconomic deprivation, and disruptions to health service delivery hinder polio immunization efforts. Although considerable attention has been devoted to logistical and health system barriers affecting vaccination uptake, growing evidence suggests that social and gender-related determinants play an equally important role in shaping children’s access to immunization services [6,7].

Gender norms influence health behaviours, access to resources, decision-making authority, and interactions with healthcare systems [8]. These norms are socially constructed expectations regarding the roles, responsibilities, and behaviours considered appropriate for women, men, girls, and boys within specific cultural contexts. In many low- and middle-income settings, gender norms shape household power dynamics, determine who controls financial resources, influence mobility and healthcare-seeking behaviour, and affect decisions regarding childhood health interventions [9]. Consequently, gender-related factors may influence whether children receive vaccinations, the timing of vaccination, and the likelihood of completing recommended immunization schedules.

Within many communities, mothers serve as the primary caregivers responsible for ensuring children’s health and wellbeing. However, their ability to access healthcare services and make independent decisions regarding childhood vaccination may be constrained by social norms, limited autonomy, restricted mobility, financial dependence, and male-dominated decision-making structures. Women’s empowerment, including decision-making authority, educational attainment, economic participation, and control over household resources, has been increasingly identified as an important determinant of childhood health outcomes [6]. Conversely, restrictive gender norms may reduce opportunities for women to seek vaccination services for their children, particularly in settings where permission from male household members is required before accessing healthcare [6].

Male involvement also plays a critical role in childhood immunization uptake [7]. In many settings, fathers, male elders, religious leaders, and other influential male figures serve as gatekeepers of household decision-making and community norms. Their attitudes toward vaccination may either facilitate or hinder immunization uptake. Positive male engagement can support healthcare-seeking behaviour, encourage participation in vaccination programmes, and improve trust in health services. Conversely, vaccine refusal, misinformation, mistrust, or restrictive gender norms among male decision-makers may contribute to missed vaccination opportunities and the persistence of zero-dose children [10].

The influence of gender norms extends beyond the household level and interacts with broader socioeconomic and structural determinants. Poverty, conflict, displacement, insecurity, limited transportation, weak health systems, and geographic isolation often disproportionately affect women and children. These structural barriers may compound existing gender inequalities by further restricting access to healthcare services and information. In conflict-affected and fragile settings, women may experience additional challenges related to safety concerns, mobility restrictions, disrupted health infrastructure, and reduced availability of healthcare personnel [11]. Such conditions can contribute to delayed, incomplete, or absent vaccination among children.

Education and access to information further shape immunization behaviours and vaccine acceptance. Maternal education has frequently been associated with improved health literacy, greater utilization of healthcare services, and increased likelihood of childhood vaccination [12]. Similarly, awareness of vaccine-preventable diseases, understanding of vaccination schedules, and trust in healthcare providers influence parental decisions regarding immunization. In contrast, misinformation, rumours, religious misconceptions, and vaccine hesitancy may undermine confidence in vaccination programmes and contribute to missed and zero-dose status among children [13]. The effectiveness of health systems in communicating with communities, addressing concerns, and providing culturally responsive services therefore remains central to improving vaccination uptake.

Although previous studies have examined determinants of childhood immunization and barriers to polio vaccination [14], evidence specifically addressing the role of gender norms in shaping missed and zero-dose vaccination remains fragmented across countries and disciplines. Existing literature includes studies examining women’s empowerment, maternal autonomy, male decision-making authority, gender-related barriers to healthcare access, vaccine hesitancy, health system responsiveness, and structural determinants of immunization [14]. However, few reviews have synthesized these diverse strands of evidence to provide a comprehensive understanding of how gender-related factors influence vaccination outcomes in countries facing persistent polio-related challenges.

Furthermore, much of the available evidence has focused on individual determinants while inadequately considering interactions among household gender dynamics, community norms, structural inequalities, and health system factors. Understanding these interconnected influences is essential for designing effective, gender-responsive strategies capable of reaching missed and zero-dose children. Such strategies are increasingly recognized as critical components of polio eradication efforts and broader immunization strengthening initiatives.

This review therefore seeks to synthesize evidence on how gender norms and gender-related social determinants influence missed and zero-dose polio vaccination among children under five years of age in Afghanistan, Nigeria, Pakistan, and Sudan. Specifically, the review explores the role of women’s empowerment and autonomy, male involvement and gender norms, socioeconomic and structural barriers, and education, awareness, and health system responsiveness in shaping vaccination access and uptake. By examining evidence across these four countries, the review aims to generate a comprehensive understanding of the mechanisms through which gender influences immunization outcomes and to identify implications for policies and programmes seeking to reduce vaccination inequities and accelerate progress toward polio eradication.

## 2. Methods

### 2.1. Study Design

This study employed a narrative systematic review design [15] to synthesize evidence on the influence of gender norms and gender-related social determinants on missed and zero-dose polio vaccination among children under five years of age in Afghanistan, Nigeria, Pakistan, and Sudan. A narrative synthesis approach was selected because the available literature comprised diverse study designs, including qualitative studies, quantitative surveys, mixed-methods investigations, programme evaluations, policy analyses, and implementation research. The heterogeneity of study methodologies, outcome measures, and contextual settings limited the feasibility of conducting a meta-analysis and necessitated an approach capable of integrating findings across multiple forms of evidence.

The review sought to develop a comprehensive understanding of the pathways through which gender-related factors influence childhood vaccination uptake and contribute to the persistence of missed and zero-dose children. Particular attention was given to exploring how household decision-making dynamics, women’s empowerment, male involvement, socioeconomic inequalities, educational factors, and health system responsiveness interact to shape vaccination outcomes in countries facing persistent challenges in polio eradication.

### 2.2. Review Question

The review was guided by the following question: How do gender norms and gender-related social determinants influence missed and zero-dose polio vaccination among children under five years of age in Afghanistan, Nigeria, Pakistan, and Sudan?

### 2.3. Eligibility Criteria

Studies were selected according to predefined inclusion and exclusion criteria developed to ensure consistency and relevance to the review objectives.

### 2.4. Inclusion Criteria

Studies were included if they examined childhood immunization, polio vaccination, routine immunization, or zero-dose vaccination among children under five years of age and investigated gender-related factors influencing vaccination uptake. These factors included, but were not limited to, women’s empowerment, maternal autonomy, male involvement, gender norms, household decision-making, and gender-based barriers to healthcare access. Eligible studies were conducted in Afghanistan, Nigeria, Pakistan, or Sudan and reported primary empirical findings using qualitative, quantitative, or mixed-methods research approaches published between 2005 and 2026. Studies were also required to include information relevant to missed vaccination opportunities, incomplete vaccination, non-vaccination, or zero-dose children. To ensure the inclusion of credible and accessible evidence, only studies published in peer-reviewed journals or reputable institutional reports, and available in English, were considered for inclusion in the review.

### 2.5. Exclusion Criteria

Studies were excluded if they focused exclusively on diseases other than polio or routine childhood immunization without relevance to vaccination uptake, examined adult vaccination programmes, or did not address gender-related determinants or factors influencing vaccination access. Editorials, commentaries, conference abstracts, opinion papers, and unpublished manuscripts lacking empirical data were also excluded, as were studies conducted outside Afghanistan, Nigeria, Pakistan, and Sudan. Studies were not excluded based on their methodological approach, as the review sought to capture diverse forms of evidence relevant to this complex phenomenon. Although a formal quality appraisal was not undertaken, methodological rigor, transparency, and the credibility of study findings were considered during data extraction and narrative synthesis. Studies providing insufficient methodological detail to interpret their findings or assess their relevance to the review objectives were excluded.

### 2.6. Information Sources

A comprehensive literature search was undertaken across multiple electronic databases to identify relevant studies. The databases searched included PubMed/MEDLINE, Scopus, Web of Science, Embase, CINAHL, Global Health, and Google Scholar. To ensure comprehensive coverage of the available evidence, the reference lists of all eligible studies were manually screened to identify additional relevant publications. The search was further supplemented through targeted searches of reports and publications produced by international organizations, public health agencies, and immunization partners working in polio-endemic and high-risk settings, thereby capturing both peer-reviewed and high-quality grey literature relevant to the review objectives.

### 2.7. Search Strategy

The search strategy combined keywords and controlled vocabulary related to gender, childhood vaccination, and the four countries of interest. Search terms were adapted to the indexing requirements and syntax of each electronic database to maximize retrieval of relevant studies. The search incorporated terms describing the population (children under five years of age, infants, young children, and zero-dose children), the exposures or determinants of interest (gender norms, gender inequality, women’s empowerment, maternal autonomy, female decision-making, male involvement, household decision-making, and gender-related barriers), and the outcomes (polio vaccination, immunization, vaccine uptake, missed vaccination, zero-dose vaccination, vaccination coverage, and immunization completion). Geographic search terms included Afghanistan, Nigeria, Pakistan, and Sudan. These concepts were combined using Boolean operators (AND/OR) to identify studies examining the influence of gender-related factors on childhood polio vaccination and immunization uptake within the selected countries. A typical search string included: (“gender norms” OR “gender inequality” OR “women’s empowerment” OR “maternal autonomy” OR “male involvement” OR “household decision making”) AND (“polio vaccination” OR immunization OR vaccine uptake OR zero-dose OR missed vaccination) AND (Afghanistan OR Nigeria OR Pakistan OR Sudan). Searches were conducted across all selected databases and updated prior to the final analysis to ensure that the most recent eligible studies were included.

### 2.8. Study Selection

**Figure 1:**
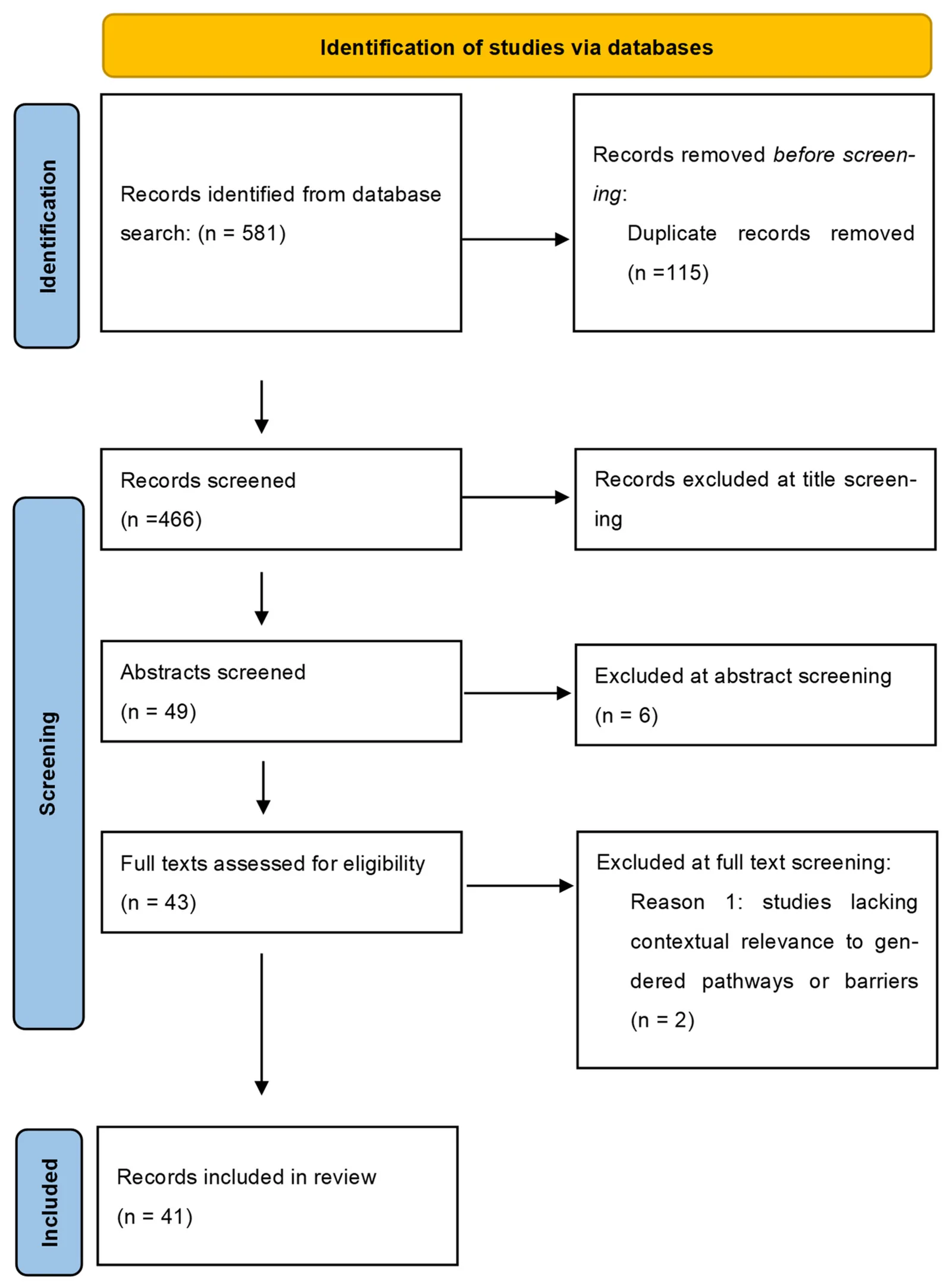
PRISMA flow chart of the selection of studies included in the analysis.

All records identified through database searching were imported into an EndNote reference management system, and duplicates were removed. Titles and abstracts were screened against the eligibility criteria. Studies considered potentially relevant proceeded to full-text review. Full-text screening was conducted to determine final eligibility. Studies meeting all inclusion criteria were retained for synthesis. Any uncertainties regarding eligibility were resolved through discussion and consensus among the review team. The studies considered for inclusion met the following criteria, as shown in Figure 1.

### 2.9. Data Extraction

A standardized data extraction form was developed to ensure consistency and accuracy in capturing relevant information from each included study. Data extracted included the author and year of publication, country of study, study design, study population, sample size, vaccination outcome examined, gender-related determinants investigated, key findings, implications for vaccination uptake, and relevance to the review objectives. Extracted data were systematically recorded in a study characteristics table to facilitate comparison across studies, and the table served as the basis for the subsequent narrative thematic synthesis.

### 2.10. Quality Assessment

A formal methodological quality assessment or risk of bias appraisal was not undertaken in this review. This decision was consistent with the narrative review approach, which aimed to provide a comprehensive synthesis of diverse forms of evidence rather than to quantitatively compare study quality or estimate pooled effects. The included literature comprised heterogeneous study designs, including qualitative studies, cross-sectional surveys, mixed-methods research, programme evaluations, implementation studies, and policy analyses, making the application of a single standardized quality appraisal tool inappropriate.

Rather than excluding studies based on methodological quality, all eligible studies that met the predefined inclusion criteria were retained to capture the breadth and complexity of evidence on gender norms and gender-related social determinants influencing missed and zero-dose polio vaccination in Afghanistan, Nigeria, Pakistan, and Sudan. Emphasis was placed on identifying recurring patterns, areas of convergence and divergence, and contextual insights across studies. Nevertheless, methodological characteristics, study design, and the consistency of findings were considered during interpretation to ensure that conclusions reflected the overall strength and coherence of the available evidence.

### 2.11. Data Synthesis

A narrative thematic synthesis approach [15] was employed to integrate findings across studies. The synthesis aimed to identify recurring patterns, relationships, and explanatory mechanisms linking gender norms to vaccination outcomes. The synthesis process involved three stages. First, findings from all included studies were reviewed repeatedly to achieve familiarity with the evidence base. Second, study findings were coded inductively to identify recurring concepts related to gender and vaccination. Similar codes were grouped into broader categories reflecting common themes across studies. Third, categories were examined and refined to generate higher-order analytical themes that explain how gender-related factors influence missed and zero-dose vaccination. These emerging themes formed the analytical framework used to organize and present the findings of the review.

### 2.12. Reporting of Findings

The findings are presented narratively according to the four thematic domains identified through the synthesis process. Within each theme, evidence from the included studies is integrated to highlight common patterns, contextual differences, and mechanisms through which gender-related factors influence vaccination uptake. Where relevant, comparisons are made across Afghanistan, Nigeria, Pakistan, and Sudan to illustrate similarities and differences in how gender norms shape childhood immunization outcomes. Particular emphasis is placed on identifying implications for policy, programme design, and gender-responsive strategies aimed at reducing the prevalence of missed and zero-dose children and strengthening polio eradication efforts.

## 3. Results

A total of 41 studies met the inclusion criteria and were included in the review. Figure 1 presents the summary of search results and screening stages. The included articles were conducted across four polio-endemic or previously endemic countries: Afghanistan, Nigeria, Pakistan, and Sudan. Of the included studies, ten were conducted in Afghanistan [16,17,18,19,20,21,22,23,24,25], 19 in Nigeria [22,25,26,27,28,29,30,31,32,33,34,35,36,37,38,39,40,41,42], ten in Pakistan [18,22,43,44,45,46,47,48,49,50] and six in Sudan [51,52,53,54,55,56].

The evidence base comprised a diverse range of study designs, including cross-sectional surveys [19,37,41,44,50,53,54], qualitative studies [21,25,26,30,32,33,36,38,49,56], mixed-methods studies [31,55], secondary data analyses of national survey datasets [16,20,22,24,27,28,40,42,43,45,46], case–control studies [18,47], reports from international agencies [17,29,34,35,51,52], two book chapters [23,48], a preprint [38] and a thesis [39]. Table 1 presents the characteristics of the included studies.

Most studies explored determinants of childhood immunization uptake, vaccine hesitancy, gender-related barriers to vaccination, maternal empowerment, health system factors, and social determinants of vaccine access. Several studies specifically addressed polio vaccination, while others examined routine childhood immunization more broadly but included findings relevant to polio vaccination and zero-dose children.

The thematic synthesis identified five overarching domains through which gender-related social determinants influenced missed and zero-dose polio vaccination among children under five years of age:

1. Women’s empowerment, autonomy and decision-making
2. Male involvement, household gender norms and family power relations
3. Socioeconomic and structural gender-related barriers
4. Education, information access, community perceptions and health system responsiveness
5. War, insurgency and their gendered impact on childhood polio immunization

**Figure 2:**
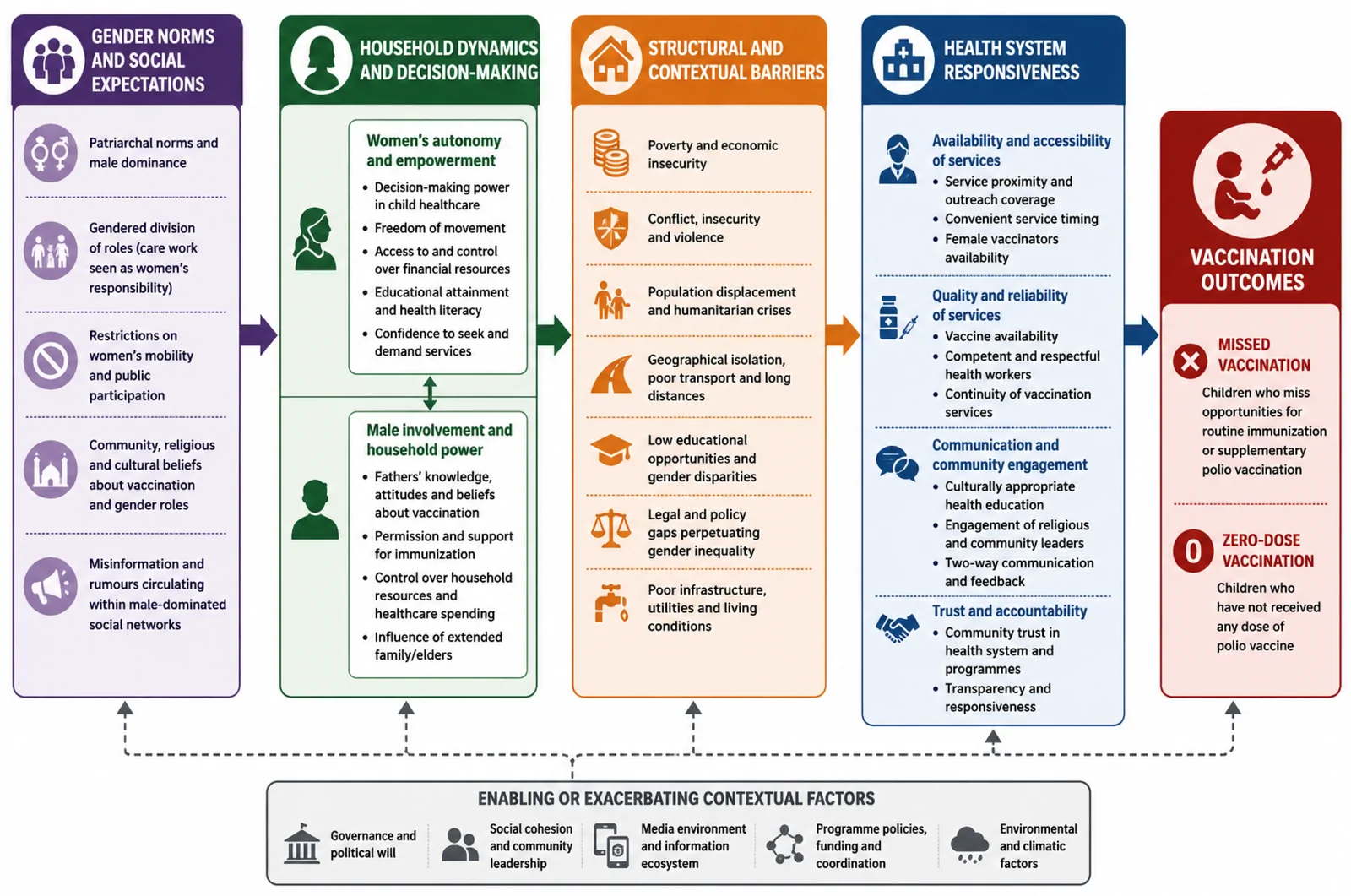
Gender-Related Social Determinants Influencing Missed and Zero-Dose Polio Vaccination.

Although these themes are presented separately, considerable overlap was observed between them. Women’s limited autonomy frequently intersected with restricted mobility, financial dependence and lower educational attainment. Similarly, community gender norms influenced household decision-making, health-seeking behaviour and trust in vaccination programmes. Structural challenges, including insecurity, conflict, displacement and inadequate health services, often amplified existing gender inequalities, disproportionately affecting women and children living in marginalized communities. Overall, the evidence suggests that gender influences childhood polio vaccination through interconnected pathways operating at individual, household, community and health system levels. Rather than functioning solely as a demographic characteristic, gender shapes access to resources, decision-making authority, mobility, exposure to health information and interactions with vaccination services. These mechanisms ultimately determine whether children receive routine immunization, supplementary polio vaccination or remain completely unvaccinated. Table 2 and Figure 2 present a summary of the gender-related social determinants and pathways influencing missed and zero-dose children identified in this review.

1. Women’s empowerment, autonomy and household decision-making

Women’s empowerment emerged as one of the most consistently reported determinants of childhood polio vaccination across the included studies. Evidence from all four countries demonstrated that mothers’ ability to make independent decisions regarding healthcare, exercise freedom of movement, access financial resources and participate in household decision-making substantially influenced whether children received timely vaccination. Importantly, the review found that empowerment should not be viewed as an isolated characteristic of individual women but rather as a multidimensional construct embedded within broader social, cultural and economic systems that regulate gender relations. Several studies highlighted the limited decision-making power of women regarding child health and immunization, suggesting that gendered power relations may contribute to missed or zero-dose vaccination among children [30,31,32,42].

In northern Nigeria, household decision-making regarding childhood immunization was often controlled by male heads of households. Some fathers opposed immunization altogether or prevented their wives from seeking vaccination services for their children, thereby restricting maternal autonomy in health-seeking behaviour [30]. However, these dynamics reflected more than individual opposition to vaccination. They were embedded within collectivist social structures in which men were widely regarded as household decision-makers, while older family members and community leaders also influenced health-related decisions. Men’s perspectives were themselves shaped by their educational attainment, socioeconomic circumstances, prevailing community norms, and the influence of female family members, illustrating that household power was relational rather than exclusively male driven. Similarly, women frequently relied on male elders and community leaders for guidance on immunization-related issues rather than making independent decisions. Young mothers reportedly sought advice from older men or community development structures before engaging with health workers, reflecting limited agency in accessing health information and services [31]. Several studies demonstrated that maternal autonomy was positively associated with childhood immunization uptake [24,25]. Women who participated in household decisions regarding healthcare expenditure, child wellbeing and health service utilization were consistently more likely to ensure that their children received routine and supplementary polio vaccinations. Conversely, when women required permission from their husbands or senior family members before leaving the household, vaccination opportunities were frequently missed, particularly during outreach campaigns that required caregivers to attend temporary vaccination sites or interact with vaccinators.

Qualitative evidence further illustrated that women’s decision-making authority often remained constrained by patriarchal household structures. Mothers commonly reported recognising the importance of vaccination but lacking sufficient authority to act independently when male household members opposed immunization or prioritised other family responsibilities [21,25,30]. In these circumstances, maternal knowledge alone was insufficient to ensure vaccine uptake because final decisions rested with husbands, fathers-in-law or elder male relatives.

Studies from Afghanistan described similar dynamics operating within highly restrictive sociocultural environments [16,19]. Women’s mobility outside the home was frequently governed by prevailing cultural norms that required accompaniment by a male guardian or restricted interactions with unrelated men. These restrictions reduced opportunities to access routine immunization services, particularly in rural and conflict-affected communities where female health workers were scarce. Consequently, even when vaccination services were geographically available, gender norms limiting women’s independent movement substantially reduced service utilisation.

Religious and sociocultural expectations further constrained women’s autonomy. In some communities, husbands discouraged women from interacting with healthcare providers outside the home, particularly male providers, thereby limiting access to vaccination services and information [32]. Collectively, these findings suggest that gendered household power structures may contribute to delayed, incomplete, or missed childhood immunization by limiting mothers’ authority to make healthcare decisions for their children.

Overall, the included evidence consistently demonstrates that limited maternal autonomy constitutes one of the most important gender-related determinants of childhood polio vaccination across Afghanistan, Nigeria, Pakistan and Sudan. Strengthening women’s decision-making authority, improving educational opportunities, expanding economic empowerment and reducing gender-related restrictions on mobility represent essential strategies for improving equitable vaccination coverage and reducing the number of zero-dose children in these settings.

2. Male involvement and gender norms

Beyond women’s individual autonomy, the review consistently demonstrated that household gender relations and prevailing social norms substantially shaped childhood polio vaccination across Afghanistan, Nigeria, Pakistan and Sudan. Gender norms determined not only who made decisions regarding children’s healthcare but also how vaccination programmes were perceived within families and communities. Rather than functioning as passive contextual influences, these norms actively structured access to vaccination through household authority, resource allocation, social expectations and community acceptance.

Across the included studies, fathers frequently occupied the primary decision-making role regarding healthcare expenditure, permission to seek services and acceptance of vaccination [20,26,32,38,41,44,53]. In patriarchal households, mothers commonly described themselves as responsible for childcare but lacking the authority to independently decide whether children would receive vaccination. Consequently, even where mothers possessed adequate knowledge regarding the benefits of immunization, implementation of that knowledge depended upon approval from male household members.

An important finding across the review was that male involvement should not be conceptualised solely as a barrier. Several studies demonstrated that fathers could become powerful facilitators of vaccination when appropriately engaged through culturally sensitive health promotion strategies [31,43]. Community mobilisation activities involving male religious leaders, traditional leaders and respected community representatives improved acceptance of vaccination by addressing concerns within influential male social networks. These interventions appeared particularly effective in settings where fathers traditionally controlled household healthcare decisions.

Evidence from Pakistan illustrated these dynamics particularly clearly. A report by LeDuc et al. described vaccination decisions as negotiated within extended family structures where husbands, fathers-in-law or senior male relatives exercised considerable influence over household health practices [49]. Mothers frequently reported delaying or missing vaccination because husbands were unavailable to provide permission, disagreed with vaccination campaigns or prioritised competing household responsibilities. Similarly, research from Sokoto State in Nigeria documented that some male household heads either opposed childhood immunization generally or prevented their wives from taking children for vaccination services [30]. These findings illustrate how paternal attitudes can directly affect whether children receive vaccines. Furthermore, such findings suggest that maternal education alone cannot overcome entrenched household power structures when authority remains concentrated among male family members.

Community governance structures also reflected gendered authority. Women frequently consulted older men and community leaders regarding immunization concerns, reinforcing the role of men as gatekeepers of health information and decisions [31]. Such patterns suggest that immunization acceptance is often mediated through male-controlled social structures. Furthermore, religious interpretations of vaccination were similarly influenced by predominantly male religious authorities. Misconceptions regarding the compatibility of polio vaccination with Islamic teachings were identified as a source of resistance, although health officials noted that such beliefs reflected misunderstandings of religious doctrine rather than established religious teachings [32]. Some women themselves cited the absence of explicit endorsement of immunization within religious texts as a reason for vaccine refusal [33], indicating how religiously framed gender norms may shape perceptions of vaccines.

Several studies further suggested that anti-gender religious narratives and practices reinforced these barriers by promoting traditional gender roles that restricted women’s autonomy, discouraged their interaction with healthcare providers, and reinforced male authority over child health decisions [32,33,38]. In such settings, resistance to gender equality was intertwined with vaccine acceptance, as religiously framed expectations that women remain subordinate to male household members limited their ability to independently seek immunization services or challenge misinformation regarding vaccines. These findings indicate that anti-gender religious norms may indirectly contribute to missed or zero-dose vaccination by constraining maternal agency and reinforcing patriarchal decision-making structures.

Studies from Sudan similarly highlighted the importance of household gender dynamics, particularly within humanitarian settings [53,54,55]. Displacement, conflict and economic instability often reinforced traditional gender roles by increasing dependence on male household members for financial support and security. Under these circumstances, women’s ability to access vaccination services became increasingly constrained, particularly where travel involved security risks or significant financial costs. The Sudanese literature also suggested that fathers’ perceptions regarding child health priorities influenced the allocation of scarce household resources. In contexts characterised by extreme poverty, healthcare decisions frequently reflected broader household survival strategies, with preventive services sometimes receiving lower priority than immediate economic needs [52,56]. Consequently, children’s vaccination status reflected both gendered household decision-making and structural socioeconomic constraints.

Overall, the literature indicates that male authority and broader gender norms significantly influence parental decision-making regarding childhood vaccination, potentially contributing to gender-related disparities in immunization access.

3. Socioeconomic inequities and structural barriers

Although household gender relations strongly influenced vaccination behaviour, the evidence consistently demonstrated that these dynamics were embedded within broader structural conditions that either constrained or facilitated access to immunization services. Across all four countries, socioeconomic disadvantage, conflict, insecurity, geographical isolation, weak health systems and limited service accessibility interacted with gender norms to produce cumulative barriers to vaccination. The review therefore indicates that gender-related determinants cannot be understood independently of the structural environments within which families live.

Poverty emerged as one of the most pervasive structural influences across the included studies [43]. Financial hardship affected vaccination through multiple mechanisms, including the inability to afford transportation, indirect costs associated with healthcare visits, opportunity costs arising from lost income and competing household priorities. Although routine childhood vaccination is generally provided without direct payment, indirect financial costs frequently discouraged attendance, particularly among households already experiencing severe economic insecurity.

Importantly, poverty was not gender-neutral. Women often lacked independent financial resources and depended upon male household members to provide transport costs or approve healthcare expenditures [26,27,30]. Consequently, children’s access to vaccination reflected both household economic status and women’s limited financial autonomy. Several studies observed that even modest transport costs became substantial barriers when women lacked direct control over household finances, illustrating how economic disadvantage and gender inequality reinforced one another [31,37,40].

Nomadic and migrant communities face unique challenges in accessing vaccination services. Population mobility across national borders and seasonal migration patterns make it difficult for health systems to maintain consistent contact with eligible children. Northern Hausa populations frequently move between Niger and Nigeria for seasonal work, while nomadic groups often follow migration routes that are poorly mapped and difficult for vaccination teams to track [34]. Similarly, vaccination strategies are complicated by the geographic dispersion and seasonal movement of nomadic communities [35]. A 2023 UNICEF report revealed the same challenges in Sudan [52].

Political and institutional factors also contribute to vaccine hesitancy and non-vaccination. In some settings, resistance to polio vaccination has been linked to broader dissatisfaction with government services. Parents were reported to reject vaccination not necessarily because of concerns about vaccine safety but as a means of expressing frustration with a health system perceived as neglectful and unresponsive to community needs [34]. Similarly, some observers suggested that polio vaccination became entangled in wider political disputes and resource allocation debates, reducing public trust in vaccination programmes [36]. These findings suggest that missed and zero-dose vaccination status may result not only from individual- or household-level decisions but also from systemic barriers, including geographic isolation, population mobility, limited service delivery reach, and political mistrust.

Geographical accessibility represented another recurring structural challenge. Rural residence, poor road infrastructure and long travel distances consistently reduced vaccination coverage across all four countries [32,35,50]. These barriers disproportionately affected women because cultural norms often restricted independent travel or required male accompaniment to visit distant health facilities. Consequently, identical geographical distances imposed substantially greater access barriers for women than for men.

Collectively, the evidence indicates that socioeconomic and structural barriers amplify existing gender inequalities rather than operating independently. Poverty, insecurity, and conflict reduce opportunities for all households to access immunization, but their effects are disproportionately experienced by women because of existing constraints on mobility, financial independence and decision-making authority. Consequently, reducing zero-dose prevalence requires interventions that address both structural inequities and gender relations simultaneously.

4. Education, awareness, and health system responsiveness

The final theme identified through the narrative synthesis concerned the interaction between education, access to health information, community perceptions and health system responsiveness. Although these determinants are often examined independently within immunization research, the included studies demonstrated that they are closely intertwined and strongly influenced by prevailing gender norms. Across all four countries, women’s access to information, their ability to interpret health messages and their interactions with health services were mediated by educational opportunities, household gender relations and broader sociocultural contexts.

Maternal education emerged as one of the most consistent predictors of childhood vaccination across the reviewed literature [22,28,39,42,43]. Mothers with higher educational attainment were generally more knowledgeable about vaccine-preventable diseases, understood recommended immunization schedules and demonstrated greater confidence in interacting with healthcare providers. Education also improved women’s capacity to identify misinformation, seek clarification from health workers and advocate for their children’s healthcare within the household. However, the evidence consistently demonstrated that education alone was insufficient to eliminate gender-related barriers to vaccination. Several studies found that educated mothers continued to experience restricted mobility, limited decision-making authority and financial dependence on male household members [22,43]. These findings indicate that educational interventions, although essential, cannot fully overcome structural gender inequalities when women remain unable to independently access health services or implement healthcare decisions.

Knowledge gaps, misinformation, and the responsiveness of health systems emerged as important determinants of vaccine acceptance and uptake. Several studies documented misconceptions surrounding polio vaccination. In northern Nigeria, resistance was linked to misunderstandings regarding the compatibility of vaccination with Islamic beliefs [32]. Some community members questioned vaccination because they did not perceive support for immunization within religious teachings [33]. Such findings highlight the role of health literacy and culturally appropriate communication in shaping vaccination decisions. Across Afghanistan and Pakistan, women’s opportunities to receive vaccination information were frequently constrained by cultural norms limiting public participation and social interaction outside the household [16,19,20,22,44,46]. Health education messages delivered through conventional public communication channels therefore often failed to reach women directly. Instead, women frequently depended on husbands, older family members, female community health workers, or informal social networks for health information. This indirect communication pathway increased the risk that misinformation or incomplete knowledge would influence vaccination decisions.

Concerns regarding vaccine safety also influenced acceptance. Persistent rumours linked polio vaccination to infertility, family planning, and population control efforts. Health officials reported continued efforts to counter these beliefs through community engagement and dialogue, particularly among Muslim populations [32]. These misconceptions may reduce demand for vaccination and contribute to missed or zero-dose status among children. Although these misconceptions affected both men and women, their influence was shaped by gendered patterns of communication and authority. In many communities, fathers and male religious leaders served as primary gatekeepers of information regarding vaccination, while mothers often lacked opportunities to independently verify conflicting messages.

In settings where communities perceived vaccination campaigns as externally driven or disconnected from broader healthcare needs, resistance was more likely to emerge despite widespread awareness of polio [43]. Health system responsiveness therefore played a critical role in shaping caregiver confidence. Communities often relied on trusted local leaders and community structures to obtain information about immunization, suggesting that health workers alone may not be sufficient sources of influence [31]. In addition, challenges in reaching nomadic populations indicate limitations in service delivery models that fail to accommodate mobile populations [34,35]. The availability of female health workers emerged as a particularly important facilitator of vaccination in conservative communities. Across Afghanistan, Pakistan and parts of northern Nigeria, women frequently expressed greater willingness to engage with female vaccinators because of prevailing cultural norms governing interactions between unrelated men and women [17,20]. Female community health workers also played important roles in providing culturally appropriate health education, identifying zero-dose children and maintaining communication with households that might otherwise remain unreached.

The literature further reveals that community resistance may reflect broader concerns regarding health system performance. Where communities perceive health services as inadequate or unresponsive, confidence in vaccination programmes may decline [34]. Strengthening trust, improving communication strategies, and adapting services to local contexts may therefore be critical for reducing missed and zero-dose vaccinations. Overall, the evidence indicates that access to accurate information is mediated through gendered social structures rather than determined solely by educational attainment. Improving vaccination coverage, therefore, requires integrated strategies that simultaneously strengthen women’s access to information, engage male decision-makers, enhance community trust, and improve health system responsiveness.

5. War, insurgency and their gendered impact on childhood polio immunization

Across Afghanistan, Pakistan, Nigeria and Sudan, armed conflict, insurgency and political instability emerged as important structural determinants of childhood polio immunization that interacted with existing gender inequalities to create additional barriers to vaccine access. Rather than acting solely through the destruction of health infrastructure, conflict influenced immunization by restricting population movement, disrupting health service delivery, displacing communities, increasing insecurity for health workers and reinforcing gender norms that constrained women’s access to healthcare and decision-making [16,18,20,25,34].

Afghanistan provided the strongest evidence of the complex relationship between conflict and immunization. Early analyses demonstrated that districts affected by prolonged conflict consistently experienced lower childhood immunization coverage than more secure areas because insecurity limited access to health facilities and disrupted routine vaccination services [23]. Decades of armed conflict weakened the health system, reduced the availability of female healthcare workers and restricted outreach activities, particularly in remote and contested provinces [16,23]. Political instability also interrupted house-to-house vaccination campaigns, leaving many children inaccessible for repeated vaccination rounds [3]. Although recent evidence suggests improvements in campaign access following political changes in Afghanistan, important geographical and gender-related barriers remain, particularly in communities where insecurity continues to limit movement and service delivery [16].

Conflict also amplified gender inequalities that affected childhood immunization. In insecure settings, women experienced greater restrictions on mobility because of concerns regarding personal safety, cultural expectations and the requirement for male accompaniment when travelling outside the home [16]. Where female health workers were unavailable, conservative gender norms further limited women’s interaction with male vaccinators, reducing opportunities for children to receive vaccination during both routine and supplementary immunization activities. Qualitative evidence further suggests that insecurity reinforced household dependence on male decision-makers, thereby reducing women’s autonomy to seek immunization services independently [26,32].

Studies from Pakistan similarly demonstrated that insecurity substantially affected vaccination coverage. Areas experiencing armed conflict and militant activity reported lower vaccination rates and higher polio incidence because vaccination teams encountered restricted access, security threats and community mistrust [43,46]. Although the nature of insecurity differed across settings, studies from Nigeria also demonstrated that insurgency and conflict complicated immunization efforts [34,35]. Insecurity in northern Nigeria limited access to underserved populations, interrupted outreach activities and increased the difficulty of reaching children living in conflict-affected or displaced communities. Conflict interacted with pre-existing gender norms by increasing dependence on male household members for movement, financial resources and healthcare decisions, thereby compounding barriers already faced by women seeking immunization services for their children [26].

Evidence from Sudan further illustrates how humanitarian crises can undermine childhood immunization. Ongoing armed conflict has contributed to widespread population displacement, destruction of health infrastructure, interruptions in vaccine supply chains and declining routine immunization coverage [51,56]. Crisis conditions disproportionately affected women, who frequently assumed primary caregiving responsibilities while simultaneously facing increased economic hardship, insecurity and restricted access to healthcare facilities [52]. Studies conducted in conflict-affected states described the need for adaptive vaccination strategies, including mobile outreach services, community engagement and targeted identification of zero-dose children to overcome barriers created by displacement and insecurity [56].

Overall, the evidence indicates that war and insurgency do not simply reduce vaccination coverage through health system disruption alone. Rather, conflict amplifies existing gender inequalities by restricting women’s mobility, reducing their access to healthcare providers, limiting the availability of female vaccinators, increasing dependence on male household decision-makers and weakening trust in health services. These intersecting effects create conditions in which children, particularly those living in conflict-affected and humanitarian settings, face an increased risk of missed or zero-dose polio vaccination. The findings suggest that equitable immunization strategies in fragile settings should integrate gender-responsive programming with conflict-sensitive service delivery, including protection of frontline health workers, expansion of female community health workers, mobile vaccination services and community engagement approaches that address both insecurity and gender-related barriers.

## 4. Discussion

This narrative review identified consistent pathways through which gender-related determinants influence missed and zero-dose polio vaccination among children in Afghanistan, Nigeria, Pakistan, and Sudan. Despite differences in sociopolitical context, health system capacity, and security conditions, gender inequalities emerged as cross-cutting determinants of childhood immunization. Across all four countries, women’s autonomy, household decision-making, access to resources, and structural barriers shaped whether children received routine and supplementary polio vaccination.

Women’s autonomy was the most consistently reported determinant. Mothers with higher education, greater decision-making authority, independent access to financial resources, and increased mobility were more likely to complete their children’s recommended vaccinations. Conversely, limited autonomy restricted immunization through dependence on male household members, mobility constraints, and reduced ability to seek services independently. These findings align with evidence from India [57] and Bangladesh [58,59], where maternal empowerment and education improved childhood vaccination completion, and with broader evidence from South Asia and Africa [60] showing that household gendered power relations remain a major barrier to equitable immunization coverage.

Although women’s autonomy was important across all countries, its effects varied by context. In Afghanistan, conflict, insecurity, and mobility restrictions intensified gender inequalities and limited access to vaccination services. Women faced overlapping barriers, including unsafe travel, limited freedom of movement, and reduced service availability in conflict-affected areas. Similar patterns have been reported in Yemen [61] and other humanitarian settings [62,63], where armed conflict interacts with restrictive gender norms to reduce immunization access. Evidence from conflict-affected regions of Ethiopia likewise shows that maternal education, distance to health facilities, and insecurity increase the likelihood of zero-dose status.

Pakistan showed similar patterns of restricted female autonomy, with greater emphasis on household decision-making, community perceptions, and vaccine misinformation. Reviewed studies consistently showed that patriarchal family systems position fathers and senior male relatives as key decision-makers for child health. Vaccine acceptance therefore often depended on male approval and wider community attitudes, not maternal preferences alone. This suggests that interventions focused only on mothers may be insufficient where fathers and community leaders strongly influence healthcare decisions.

In Nigeria, gender-related barriers intersected with socioeconomic disadvantage, educational inequality, and health system limitations. Although women’s autonomy predicted immunization uptake, poverty and male control over household resources often constrained mothers’ ability to access vaccination services. Recent Nigerian evidence similarly shows that women’s participation in household and healthcare decision-making improves childhood vaccination coverage, although effects vary by geography and local sociocultural norms. These findings support the Cooper et al. qualitative evidence synthesis, which identified social exclusion as a key mechanism reducing childhood vaccination acceptance in low- and middle-income countries [64]. In this review, the intersection of gender inequality, poverty, and weak health systems suggests that under-immunization often reflects structural disadvantage rather than knowledge deficits alone.

Sudan presented a humanitarian context in which gender inequalities were compounded by conflict, displacement, and health system disruption. Women assumed greater caregiving responsibilities during instability while facing reduced access to services because of insecurity, population displacement, and damaged health infrastructure. Similar patterns have been documented in conflict-affected populations across the Middle East and North Africa [65], where displacement, poverty, mobility constraints, limited transport, and weakened health systems disproportionately affect women and children and contribute to persistent zero-dose status.

A key finding is that gender-related determinants operate through interconnected pathways rather than as isolated risk factors. Women’s limited agency often coexisted with poverty, low education, geographical isolation, insecurity, and weak health systems, supporting socioecological models that emphasize multiple levels of influence on healthcare utilization. Similar conclusions have been reported from Ethiopia, where maternal education, healthcare accessibility, and social inequities collectively contribute to zero-dose status [66]. The findings also align with Cooper et al., who argue that vaccine acceptance is shaped by broader social and political contexts, including social exclusion [64]. Across the four countries, barriers such as conflict, gender discrimination, poverty, poor service quality, and exclusion from decision-making reduced both access to services and confidence in vaccination programmes. Integrated approaches addressing gender norms and structural barriers are therefore needed to reach persistently underserved populations.

This review supports making gender-transformative approaches central to immunization strategies in polio-endemic and high-risk settings. Traditional programmes have prioritized vaccine supply and service delivery but have often paid less attention to the social and gendered factors shaping uptake. Improving women’s decision-making power, expanding girls’ and women’s education, engaging men as supportive partners, reducing mobility restrictions, and strengthening gender-responsive health systems could reduce missed and zero-dose vaccination. In conflict-affected settings, women-led community mobilization, female health workers, mobile vaccination services, and engagement with religious and traditional leaders may improve access and acceptance among marginalized populations.

### Policy Implications and Recommendations

Gender-responsive policies should be embedded within national polio eradication and routine immunization strategies rather than implemented as stand-alone interventions. Policymakers should strengthen women’s decision-making capacity through girls’ education, women’s economic empowerment, and equitable participation in household health decisions. Because mothers rarely decide alone, programmes should also engage fathers, male household heads, religious leaders, and other community gatekeepers through culturally appropriate interventions that promote shared responsibility for child health and challenge harmful norms restricting women’s autonomy and vaccination access.

Gender-related barriers are inseparable from broader structural inequities, including poverty, weak health systems, geographical isolation, political mistrust, and conflict. Policymakers should adopt intersectional, equity-focused strategies that prioritize underserved populations. This includes expanding mobile vaccination services, strengthening outreach through female community health workers, improving transport and service access for rural and nomadic communities, and integrating immunization with primary healthcare and social protection services. In humanitarian settings, policies should use conflict-sensitive and gender-responsive approaches that protect frontline health workers, sustain vaccine delivery during insecurity, and reach displaced and hard-to-reach populations through flexible service models.

Improving vaccine uptake also requires rebuilding trust between communities and health systems. Policymakers should move beyond information campaigns that only correct misinformation and address underlying drivers of hesitancy, including social exclusion, poor-quality services, and limited community participation in programme design. Sustained engagement with local leaders, women’s groups, religious institutions, and civil society organizations can strengthen community ownership and ensure that interventions respond to local gender norms and sociocultural contexts. Routine monitoring should include gender-sensitive indicators such as women’s decision-making autonomy, male involvement, access to female vaccinators, and gender-related barriers to service use.

The findings should be interpreted in light of the diverse geopolitical, cultural, and health system contexts of the included countries. Although Afghanistan, Nigeria, Pakistan, and Sudan share challenges related to polio eradication, conflict, and inequitable immunization coverage, differences in governance, security, sociocultural norms, and health service delivery may limit the transferability of findings and the extent to which direct cross-country comparisons can be made.

## 5. Conclusions

Overall, despite contextual variation across Afghanistan, Pakistan, Nigeria, and Sudan, the evidence shows a clear convergence in how gender influences childhood polio immunization. Gender operates through intersecting household, community, structural, and health-system pathways. Family power relations are more complex than male decision-making alone: while men often act as the final decision-makers, their choices are shaped by maternal education and by female family members, including mothers and mothers-in-law, who may reinforce or challenge patriarchal norms. Gender-transformative strategies should therefore strengthen women’s agency while constructively engaging men through culturally appropriate approaches such as husband schools. Together with trusted community leadership, female health workers, and context-responsive services, these strategies can increase vaccine uptake among missed and zero-dose children and accelerate polio eradication.

## Figures and Tables

**Table 1:** Characteristics of the included studies.

First Author, Year	Country	Source Type/Study Design	Sample Size	Population	Themes
Mashal, 2007 [16]	Afghanistan	Secondary data analysis	331 districts	Children under 12	3, 4 and 5
UNICEF, 2021 [17]	Afghanistan	Report	-	-	3, 5
O’Reilly, 2012 [18]	Afghanistan, Pakistan	Secondary data analysis	46,977	Children aged 0–14	5
SteelFisher, 2017 [19]	Afghanistan	Primary study	1980	Caregivers of children under 5	3, 5
Sabawoon, 2024 [20]	Afghanistan	Secondary data analysis	-	-	3, 5
Sillab, 2025 [21]	Afghanistan	Primary study	22	Families who refused vaccination	4, 5
Muzammil, 2021 [22]	Afghanistan, Pakistan, Nigeria	Secondary data analysis	60,118	Ever-married women aged 15–49	1, 2, 5
Farr, 2018 [23]	Afghanistan	Book chapter	-	-	5
Khatir, 2025 [24]	Multi-country	Secondary data analysis	205,827	Children aged 12–23 months	1
Kalbarczyk, 2021 [25]	Multi-country	Primary study	196	Key informants in polio	1, 2
Kuss, 2026 [26]	Nigeria	Primary study		Desk review, FGD and key informants	1, 3
Singh, 2013 [27]	Nigeria	Secondary data analysis		Married women with a child aged 12–23 months	1, 2
Ayodele, 2024 [28]	Nigeria	Secondary data analysis	1713	Women aged 15–49 years	1, 4
WHO, 2018 [29]	Nigeria	Report	-	-	1, 4
Abad, 2021 [30]	Nigeria	Primary study	193	Purposively sampled informants	3, 4
Akwataghibe, 2019 [31]	Nigeria	Primary study	215	Caregivers, stakeholders and community reps	3, 4
Olufowote, 2021 [32]	Nigeria	Primary study	168		3
Murele, 2014 [33]	Nigeria	Primary study	72	Caregivers	4
Cheng, 2008 [34]	Nigeria	Report	-	-	3, 4
Gidado, 2013 [35]	Nigeria	Secondary data analysis/report	-	-	3
Ghinai, 2013 [36]	Nigeria	Secondary data analysis	-	-	4
Antai, 2012 [37]	Nigeria	Secondary data analysis	33,385	Women aged 15–49 years	1, 2
Iliyasu, 2025 [38]	Nigeria	Primary study	400	Caregivers, religious leaders, and community influencers	1, 2
Yehualashet, 2021 [39]	Nigeria	Primary study		Key informants, health facility workers	1, 2
Gayawan, 2026 [40]	Nigeria	Secondary data analysis	17,892	Mothers and children	1, 2
Chauhadry, 2024 [43]	Pakistan	Secondary data analysis	120,000	Children under 5 years	3
Khan, 2017 [44]	Pakistan	Secondary data analysis	13,558	Married women aged between 15 and 49 years	1
Joachim, 2024 [45]	Pakistan	Secondary data analysis	4726	Children aged 12–23 months	3
Verma, 2017 [46]	Pakistan	Secondary data analysis		Children (not very clear)	4
Soofi, 2023 [47]	Pakistan	Primary study	750	‘Cases’/households	3, 4
Khawaja, 2025 [48]	Pakistan	Secondary data analysis	153	Parents	1, 2
LeDuc, 2023 [49]	Multi-national	Secondary data analysis	-	-	1, 3, 4
Abbas, 2026 [50]	Pakistan	Primary study	600	Adults in rural and urban settings	3, 4
UNICEF, 2025 [51]	Sudan	Report	-	-	3, 5
UNICEF, 2023 [52]	Multi-national	Report	-	-	3
Abdallah, 2024 [53]	Sudan	Primary study	400	Parents	1, 4
Ibnouf, 2007 [54]	Sudan	Primary study	410	Children under 5	1, 3

**Table 2:** Summary of Gender-Related Social Determinants Influencing Missed and Zero-Dose Polio Vaccination.

Theme/Sub-Theme	Gender-Related Determinant	Mechanism Influencing Vaccination
Women’s empowerment	Limited household decision-making	Delayed or prevented caregiver action despite positive attitudes
Women’s mobility	Restrictions on independent travel	Reduced access to vaccination services and outreach
Economic dependence	Lack of financial autonomy	Limited ability to pay indirect vaccination costs
Education	Low maternal education	Reduced awareness of schedules and health services
Household gender norms	Male-dominated healthcare decisions	Mothers unable to independently vaccinate children
Male involvement	Supportive fathers improve uptake	Facilitated transport, permission and household acceptance
Community gender norms	Patriarchal expectations	Reinforced restrictions on maternal autonomy
Poverty	Financial constraints	Missed appointments and reduced service utilisation
Conflict and insecurity	Disrupted health services	Increased zero-dose prevalence
Population displacement	Interrupted routine immunization	Difficulty reaching mobile populations
Geographic isolation	Long travel distance	Reduced healthcare utilisation
Health system barriers	Inadequate outreach, workforce shortages	Missed opportunities for vaccination
Community trust	Confidence in health services	Greater acceptance of vaccination programmes

## Data Availability

The original contributions presented in this study are included in the article. Further inquiries can be directed to the corresponding author.
